# SLC13A4 Might Serve as a Prognostic Biomarker and be Correlated with Immune Infiltration into Head and Neck Squamous Cell Carcinoma

**DOI:** 10.3389/pore.2021.1609967

**Published:** 2021-11-10

**Authors:** Meng-Ling Yang, Jia-Hua Zhang, Sheng Li, Rui Zhu, Li Wang

**Affiliations:** ^1^ Department of Integrated Traditional Chinese and Western Medicine, Union Hospital, Tongji Medical College, Huazhong University of Science and Technology, Wuhan, China; ^2^ Center for Stem Cell Research and Application, Union Hospital, Tongji Medical College, Huazhong University of Science and Technology, Wuhan, China; ^3^ Department of General Surgery, Hospital of Huazhong University of Science and Technology, Wuhan, China; ^4^ Department of Emergency Surgery, Union Hospital, Tongji Medical College, Huazhong University of Science and Technology, Wuhan, China

**Keywords:** biomarker, tumor-infiltrating immune cells, SLC13A4, head and neck squamous cell carcinoma, database analysis

## Abstract

SLC13A4 is a sodium sulfate co-transporter, which is expressed in brains, placentas, thymes and other tissues, plays an essential role in maintaining the metabolic balance of sulfate *in vivo*. The TCGA database shows that it is differentially expressed in a variety of tumors, but its prognostic value in tumors has not been clarified. TCGA, Oncomine and Timer databases were used to analyze SLC13A4 mRNA expression in cancer tissues and normal tissues, and its correlation with clinical prognosis in head and neck tumor. The CIBERSORT database was used to analyze the correlation between SLC13A4 expression and the infiltration of immune cells. SLC13A4 enrichment analysis was carried out by GSEA. SLC13A4 mRNA levels were significantly lower in head and neck tumors than in paracancer tissues. SLC13A4 expression in Head and neck squamous cell carcinoma (HNSCC) was closely related to tumor pathological grade and clinical stage. Decreased SLC13A4 expression was associated with poor overall survival (OS), progression free survival (PFS), disease specific survival (DSS) and recurrence free survival (RFS) in HNSCC patients. The expression of SLC13A4 was negatively correlated with Monocytes, M1 macrophages, M2 macrophages, resting CD4+ memory T cells, resting NK cells and activated NK cells, but positively correlated with neutrophils, plasma cells, T follicular helper cells, gamma delta T cells, regulatory T cells and naive B cells. In addition, the genes in SLC13A4 low-expression group were mainly concentrated in immunity-related activities, viral diseases, typical tumor pathways and metabolism. The SLC13A4 high expression group was mainly enriched in metabolic pathways. These suggest that SLC13A4 may be a potential prognostic biomarker in HNSC and correlated with immune infiltrates.

## Introduction

Most head and neck cancers are squamous cell carcinomas that originate in the upper respiratory epithelia (located in oral cavity, oropharynx, larynx or hypopharynx). Head and neck squamous cell carcinoma (HNSCC) tend to develop in the mucous membrane of the mouth, nose, and throat. It is the sixth most common cancer around the globe, with a 5-year mortality rate of 50% [1,2]. Although the application of various treatment modalities, and combination use of surgical, radiotherapeutic and chemotherapeutic treatments can significantly reduce the short-term and long-term survival of the malignancy, the situation remains grim [3]. The immune cells and stromal infiltration contribute to the tumor microenvironment (TME), in which various immune cells act as either promoters or inhibitors of cancers, and they are closely related to the prognosis of patients [4]. Intensive researches demonstrated that PD-1 inhibitors exert a significant therapeutic effect on HNSCC [5,6]. In the clinical practice, early diagnosis and accurate prognostic prediction of HNSCC patient remain a challenge due to the lack of specific biomarkers. To improve the long-term survival of tumor patients, it is of great importance to identify the biomarkers that are conductive to early diagnosis, precise outcome prediction of cancers and to the identification of people who well respond to immunotherapy.

A feature shared by all members of solute carrier family 13 (SLC13) is the multi-bonded proteins encoding 8–13 transmembrane domains, and is found in a wide array of tissues. SLC13 family has 5 members. In terms of gene codes and function, they fall into two categories: sodium sulfate co-transporter (SLC13A1, SLC13A4) and sodium carboxylic acid co-transporter (SLC13A2, SLC13A3, SLC13A5) [7]. SLC13A4 is one of the major transporters of sulfate metabolism and plays an anti-apoptotic part by mediating the transmembrane transportion of thiosulfate on cell membrane, regulating the concentration of intracellular sulfate and modulate caspase-3 sulfurization [8]. SLC13A4 is very different from other sulfate transporters, and its functions have been preliminarily explored in tonsil, placenta, testis and central nervous system, but the physiological implication of its low-level expression in heart, thymus and liver has yet to be elucidated [9–12]. Moreover, its roles in tumors remains poorly understood. The mutated sulfate transporter gene and/or disordered sulfate metabolism are closely related to multiple human diseases, such as aberrant early embryonic development, chondrodysplasias and Hunter syndrome, which may be the predisposing factors of neurodevelopmental diseases, such as mental retardation and autism spectrum disorder [11,13–15]. Thiosulfate is one of the important inorganic sulfates in the body and the fourth most abundant anion in mammalian plasma, and is crucial for a great many physiological functions [16,17]. In prostate cancer, the content of thiosulfate is used as an auxiliary diagnostic marker [18]. All these highlight the important role that sulfate transporter protein plays in human physiology. In addition, thiosulfate is an important product of H2S metabolism. H2S was reported to induce DNA damage and affect the cell signal transduction and cell cycle, and participate in a variety of physiological and pathological processes, including tumorigenesis and development [19–21]. These further indicate that the sulfate process of metabolic pathway may be closely involved in TME.

This study examined the differential expression, clinical relevance and prognostic value of SLC13A4 in HNSCC by searching RNA-seq and clinical data in the TCGA public database. On the basis of its expression in thymus, we further analyzed the potential pathways and possible molecular mechanisms by which SLC13A4 is involved in HNSCC tumor immune infiltration by searching cibersort and GSEA database.

## Materials and Methods

### TCGA Database

All level 3 RNA expression data (Workflow Type: HTSeq-FPKM) from 502 HNSCC cancer tissues and 44 adjacent-cancer tissues were retrieved from the Cancer Genome Atlas (TCGA) database (https://tcga-data.nci.nih.gov/tcga/) [22]. The clinical information of HNSCC patients, including age at diagnosis, gender, HPV status, radiation therapy, pathological stage, T stage, N stage, M stage, grades, overall survival time, overall survival status, disease free survival time, disease free survival status, disease specific survival time, disease specific survival status, progression-free survival time, progression-free survival status were also downloaded from the TCGA. TCGA-HNSCC has 500 HNSCC patients with clinical information, but the information for each patient is not complete. In detail, TCGA-HNSCC provides gender information for 500 patients, age information for 499 patients, HPV status information for 481 patients, T stage information for 444 patients, N stage information for 407 patients, and M stage information for 187 patients, pathological stage information for 432 patients, grade information for 481 patients and radiation therapy information for 429 patients. Furthermore, 500 patients’ overall survival status information, 499 patients’ overall survival time information, progression free survival time and status, 474 patients’ disease specific survival time and status, 125 patients’ disease specific survival time and status were provided. All data are included in the study. According to the publication guidelines, datasets can be used for publication without restrictions or limitations.

### GEO Database

RNA-seq data of SLC13A4 in GSE2837, GSE27020 and GSE41613 were obtained from Gene Expression Omnibus (GEO; https://www.ncbi.nlm.nih.gov/geo/) [23] to study the relationship between SLC13A4 expression level and prognosis of HNSCC patients. Normalized expression matrix files and sequencing platform annotations of the gene sets were downloaded.

### Oncomine Database Analysis

Oncomine database (https://www.oncomine.org/resource/main.html) [24] was used for analyzing SLC13A4 expression in various cancer types. The cut-off value of *p* value and fold change were 0.01 and 2, respectively.

### TIMER Database Analysis

TIMER database (https://cistrome.shinyapps.io/timer/) [25] contained 10,897 samples, including 32 different kinds of cancer types covered by the TCGA dataset. We used the database to evaluate the SLC13A4 expression in tumors of various types and its correlation with T cell exhaustion markers.

### The Human Protein Atlas

The human protein atlas (HPA) contains the detailed RNA sequences of 37 major normal tissue types and immunohistochemical results of tissue microarray involving 44 different tissue types. HPA (https://www.proteinatlas.org) [26] has the data about the expression and localization of human proteins in tissues and organs. All the data are openly accessible. We analyzed the expression of SLC13A4 protein in normal oral tissues and oral squamous cell carcinoma by on the basis of immunohistochemistry image in HPA. The antibody used is HPA048582 (SIGMA-ALDRICH).

### Survival Analysis

Survival analysis was performed by using survival and survminer packages in R. 499 patients had detailed survival time data and progression-free survival time data, 474 patients had disease specific survival time data, and 124 patients had disease free survival time data in TCGA. 97 Patients had overall survival time data in GSE41613. 28 Patients had recurrence-free survival time data in GSE2837. 109 Patients had disease specific survival time data in GSE27020. All the time data were employed for survival analysis. The median value of survival time from TCGA database and mean value of survival time from GEO database was used as the cut-off value for the survival analysis. The survival curve was plotted by using Kaplan-Meier method, and log rank was used for significance assessment. A *p* < 0.05 was considered statistically significant.

### Immune Infiltration Analysis

To estimate the abundance profile of the tumor-infiltrating immune cells (TIICs) in HNSCC, CIBERSORT (http://cibersort.stanford.edu/) [26] computational method was applied for analyzing mRNA sequence data of all tumor samples. Based on the validated leukocyte gene signature matrix (termed LM22), the proportion of infiltration of 22 kinds of immune cells in each sample was statistically analyzed. In each sample, the ratio of all TIICs was equal to 1.

### Barplot, Corrplot, Vioplot and Scatter Plot

Barplot and corrplot were visualized by using R software with package corrplot, while vioplot was produced with packages BiocManager and vioplot. Except for package BiocManager, scatter plotting was performed by employing R software with packages ggplot2, ggpubr and ggExtra.

### Gene Set Enrichment Analysis

GSEA is supported by the Broad Institute Website (http://software.broadinstitute.org/gsea/index.jsp) [27]. We got HNSCC RNA-sequencing data from TCGA, and performed gene set enrichment analysis by using GSEA software (V4.0.2). We downloaded kegg, hallmark and immune signatures gene sets from Molecular Signatures Database to investigate the possible biological functions of SLC13A4. Gene sets with nominal *p* value < 0.05 and FDR ≤ 25% were considered statistically significant.

### Statistical Analysis

All statistical analyses were conducted by using SPSS 26.0 statistical software (SPSS, Chicago, IL), GraphPad prism 7.0, R X64 4.0.0 and Bioconductor packages (https://www.bioconductor.org/). Wilcoxon test was used to compare the different proportions of TIICs between high SLC13A4 expression group and low expression group. Fisher test was employed to analyze the relationship between SLC13A4 mRNA expression and clinical characteristics. Univariate Cox regression models were utilized to study the prognostic value of clinicopathological parameters for HNSCC. The multivariate Cox regression models were used to assess the association between SLC13A4 mRNA expression and survival adjusted for age, gender, T stage, N stage, pathological stage, grade, HPV status and radiation therapy. Overall survival (OS), disease free survival (DFS), progression free survival (PFS), disease specific survival (DSS) and recurrence free survival (RFS) curves were calculated by Kaplan-Meier method. GraphPad Prism 7.0 was applied for statistical analysis of clinical information. Normal distribution data were subjected to parametric test, and non-normal distribution data to Mann-Whitney test. Significant difference was set at *p* < 0.05.

## Results

### Expression of SLC13A4 in Various Human Cancer Types

**FIGURE 1:**
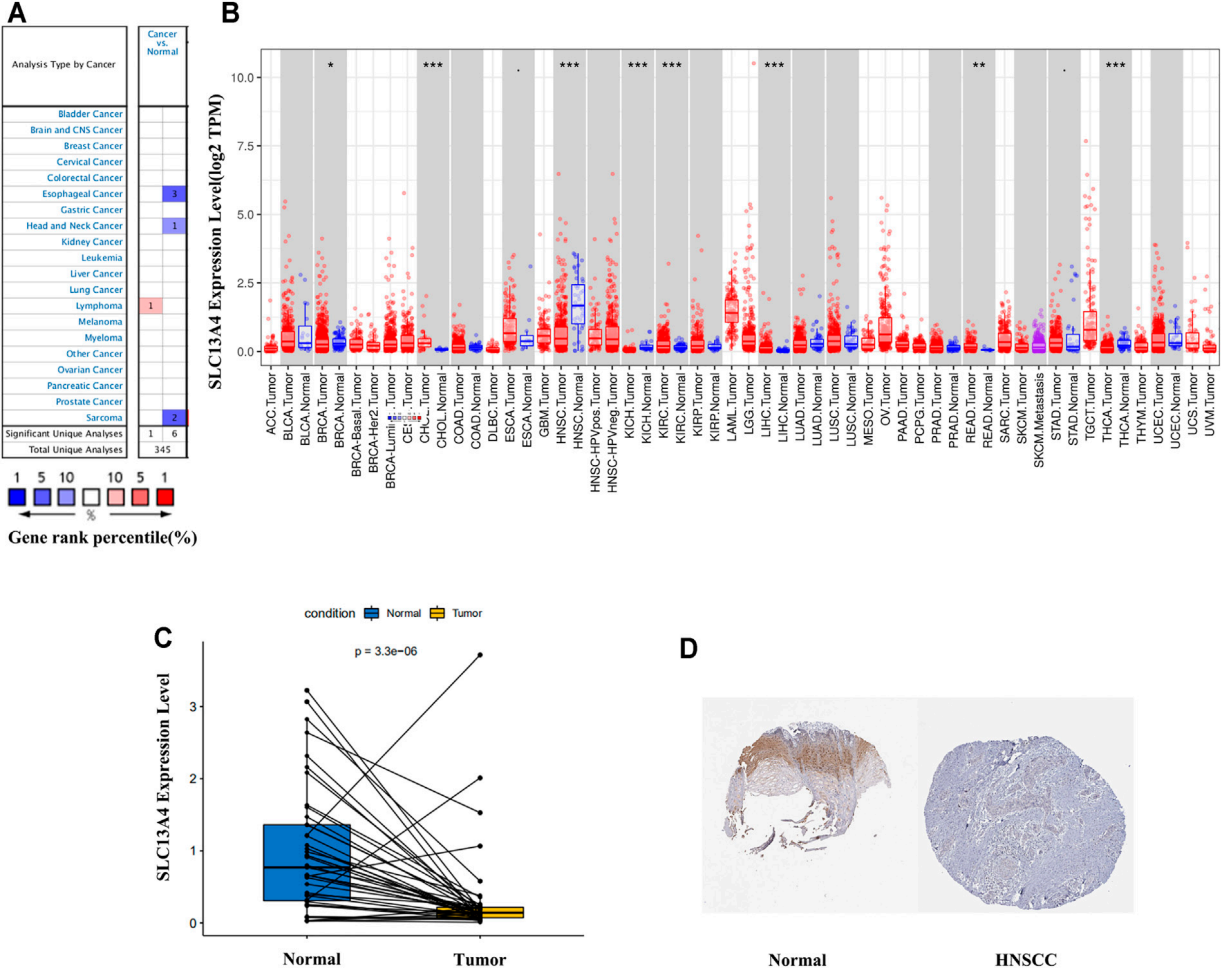
SLC13A4 expression levels in different types of human cancers. **(A)** Increased or decreased SLC13A4 in data sets of different cancers compared with normal tissues in the Oncomine database. **(B)** Human SLC13A4 expression levels in different tumor types from TCGA database were determined by TIMER (*p < 0.05, **p < 0.01, ***p < 0.001). **(C)** SLC13A4 expression levels between adjacent and tumor tissues in HNSCC patients from TCGA database. **(D)** The expression of SLC13A4 protein in normal oral tissues and oral squamous cell carcinoma was observed using immunohistochemistry *via* HPA.

Using Oncomine database, we analyzed SLC13A4 gene expression in different tumors and the adjacent tissues. The results are shown in Figure 1A. SLC13A4 expression was higher in lymphoma tissues than in adjacent tissues, and was lower in esophageal cancer, HNSCC and sarcoma. To further evaluate SLC13A4 expression in various tumors, we used Timer database to analyze the transcriptional sequence data of 22 TCGA tumors. The results are shown in Figures 1B,C. SLC13A4 expression was lower in breast cancer, head and neck squamous cell carcinoma, kidney chromophobe, thyroid cancer, and higher in renal clear cell carcinoma, hepatocellular carcinoma, cholangiocarcinoma, and rectal adenocarcinoma. In addition, immunohistochemical analyses available from the HPA are shown in Figure 1D, SLC13A4 expression was lower in tumor tissues when compared to normal tissues.

### Correlation Between SLC13A4 Expression and Clinical Features in HNSCC Patients

We obtained the data on transcription sequences and clinical characteristics of HNSCC patients from the TCGA database, and divided HNSCC patients into the high-expression group and the low-expression group in terms of the median mRNA expression of SLC13A4. The results are shown in Table 1. HPV status and overall survival in HNSCC patients were significantly correlated with SLC13A4 mRNA expression. HPV-positivity rate (17.7%) was higher in high SLC13A4 expression group than in its low-expression counterpart (10.9%). For the overall survival status, the proportion of deaths in HNSCC patients with high SLC13A4 expression (34.4%) was significantly lower than that with low SLC13A4 expression (52.4%).

**TABLE 1:** Relationship between clinical character and SLC13A4 expression in HNSCC.

**Characters**	**Level**	**Low expression of SLC13A4**	**High expression of SLC13A4**	***p*** **Value**
n		250	250	
Gender	Female	68 (27.2%)	65 (26.0%)	0.840
	Male	182 (72.8%)	185 (74.0%)	
Age	<60 years	100 (40.2%)	120 (48.0%)	0.087
	≥60 years	149 (59.8%)	130 (52.0%)	
HPV status	HPV(−)	212 (89.1%)	200 (82.3%)	0.038
	HPV(+)	26 (10.9%)	43 (17.7%)	
T Stage (%)	T1	19 (8.6%)	26 (11.6%)	0.252
	T2	67 (30.5%)	65 (29.0%)	
	T3	55 (25.0%)	41 (18.3%)	
	T4	79 (35.9%)	92 (41.1%)	
N Stage (%)	N0	72 (36.7%)	99 (46.9%)	0.192
	N1	34 (17.3%)	31 (14.7%)	
	N2	87 (44.4%)	77 (36.5%)	
	N3	3 (1.5%)	4 (1.9%)	
M Stage (%)	M0	80 (100.0%)	106 (99.1%)	0.572
	M1	0 (0.0%)	1 (0.9%)	
Pathological stage (%)	Stage I	11 (5.1%)	14 (6.5%)	0.921
	Stage II	36 (16.7%)	34 (15.7%)	
	Stage III	40 (18.6%)	38 (17.5%)	
	Stage IV	128 (59.5%)	131 (60.4%)	
Grade	G1	23 (9.5%)	38 (15.9%)	0.069
	G2	155 (64.0%)	144 (60.3%)	
	G3	64 (26.4%)	55 (23.0%)	
	G4	0 (0.0%)	2 (0.8%)	
Radiation therapy (%)	No	76 (36.2%)	74 (33.8%)	0.614
	Yes	134 (63.8%)	145 (66.2%)	
Overall survival_status	Alive	119 (47.6%)	164 (65.6%)	<0.001
	Dead	131 (52.4%)	86 (34.4%)	

**FIGURE 2:**
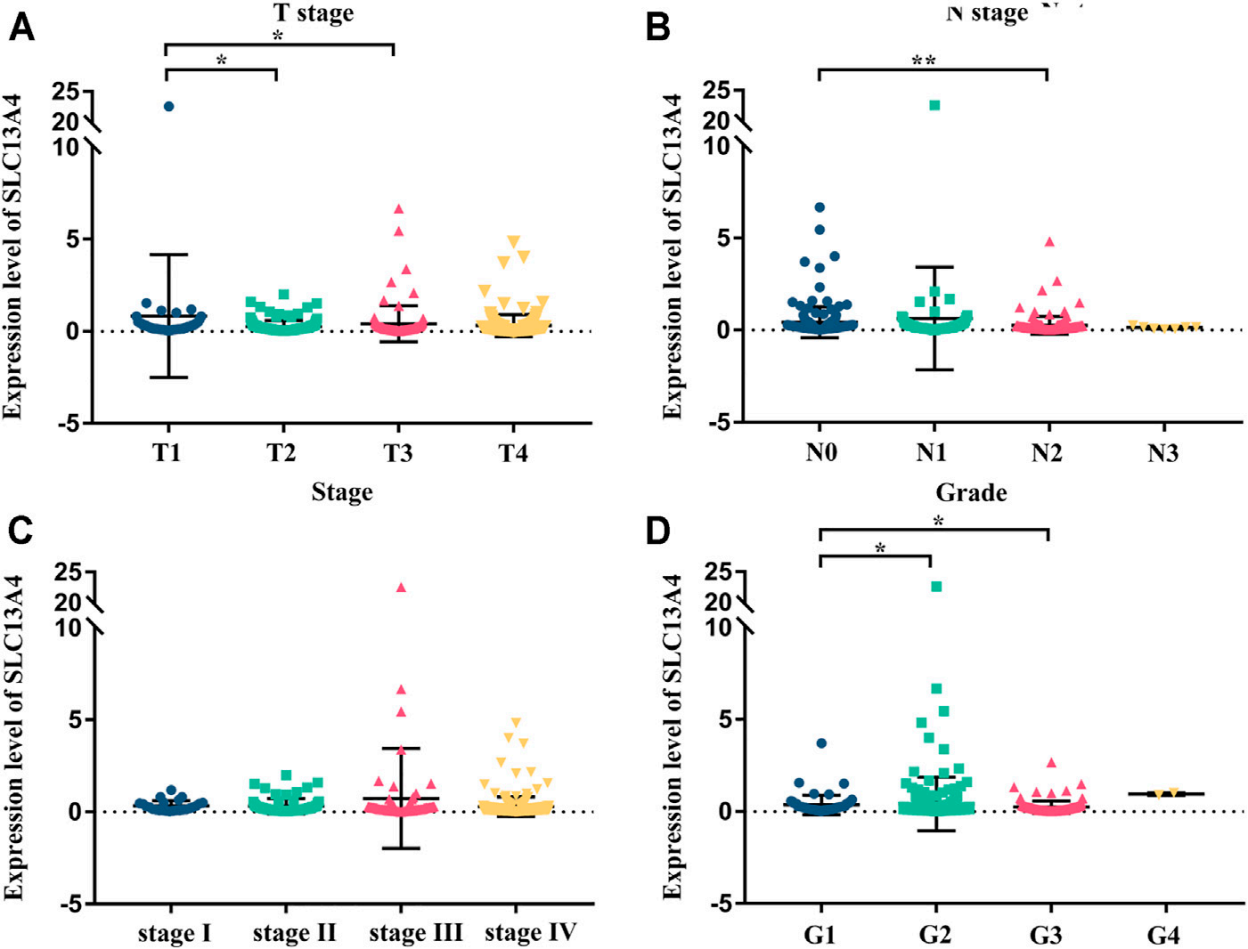
Relationship between mRNA expression of SLC13A4 and clinical characteristic in HNSCC patients. mRNA expression of SLC13A4 were remarkably correlative with T stage, N stage and grade. Patients in advanced T stage **(A)**, N stage **(B)**, clinical stage **(C)** and grade **(D)** tends to show lower mRNA expression of SLC13A4 (*p < 0.05, **p < 0.01).

We grouped the clinical characteristics of the patients and compared the differences in mRNA expression of SLC13A4 in each group. The results are shown in Figure 2 and Table 1. SLC13A4 expression showed statistically significant differences among T stage, N stage and pathological grades, exhibiting an overall tendency that the more malignant the tumor, the lower the mRNA expression of SLC13A4. The mRNA expression level of SLC13A4 at T1 stage was significantly higher than those at T2 stage (*p* = 0.0342) and T3 stage (*p* = 0.0175). SLC13A4 expression level at N0 stage was significantly higher than that at N2 stage (*p* = 0.0022). SLC13A4 expression at grade 1 was significantly higher than those at grade 2 (*p* = 0.0287) and grade 3 (*p* = 0.003). Since there were only two samples that were in grade 4, no statistical comparison was made.

### Univariate and Multivariate Cox Regression Analysis of Factors Affecting Survival in HNSCC Patients

To assess the risk factors influencing the prognosis of HNSCC patients, Cox regression models were used to analyze the clinical data of the patients. Univariate analysis revealed that SLC13A4 expression level, T Stage, N Stage and pathological stage are high risk factors affecting overall survival, disease specific survival and progression free survival in patients with HNSCC. In addition, HPV negative status is also a higher risk factor affecting both overall survival and progression free survival, and radiation therapy is a risk factor only affecting overall survival (Table 2). Moreover, after adjusted for age, gender, T Stage, N Stage, pathological stage, grade, HPV status and radiation therapy, the relationship between SLC13A4 mRNA expression and overall survival, disease specific survival and progression free survival were still significant (Tables 3–5).

**TABLE 2:** Univariate Cox regression analysis of factors affecting survival in HNSCC patients.

Characters	Overall survival		Progression free survival		Disease specific survival	
	HR (95%CI)	*p* Value	HR (95%CI)	*p* value	HR (95%CI)	*p* Value
Gender	0.751 (0.564–1.001)	0.051	1.042 (0.753–1.443)	0.802	0.956 (0.643–1.421)	0.825
Age	1.293 (0.982–1.703)	0.067	1.105 (0.830–1.472)	0.493	1.075 (0.757–1.525)	0.688
HPV status	0.503 (0.306–0.826)	0.007	0.598 (0.372–0.961)	0.034	0.569 (0.314–1.032)	0.063
T Stage	1.300 (1.126–1.501)	<0.001	1.286 (1.105–1.495)	0.001	1.374 (1.137–1.662)	0.001
N Stage	1.532 (1.295–1.813)	<0.001	1.433 (1.205–1.703)	<0.001	1.729 (1.384–2.160)	<0.001
Pathological Stage	1.416 (1.182–1.696)	<0.001	1.339 (1.114–1.608)	0.002	1.519 (1.187–1.944)	0.001
Grade	1.103 (0.895–1.358)	0.358	1.075 (0.860–1.344)	0.526	1.144 (0.870–1.503)	0.336
Radiation Therapy	0.639 (0.467–0.874)	0.005	0.893 (0.640–1.248)	0.508	0.780 (0.512–1.190)	0.250
SLC13A4	0.601 (0.457–0.789)	<0.001	0.587 (0.440–0.784)	<0.001	0.461 (0.320–0.664)	<0.001

**TABLE 3:** Relationship between SLC13A4 expression, clinical character and overall survival in HNSCC patients.

Variables	Model 1 (*n* = 499)^a^		Model 2 (*n* = 499)^b^		Model 3 (*n* = 386)^c^		Model 4 (*n* = 331)^d^	
	HR (95% CI)	*p* Value	HR (95% CI)	*p* Value	HR (95% CI)	*p* Value	HR (95% CI)	*p* Value
SLC13A4*								
Low expression group	1.665 (1.267–2.187)	*p* < 0.001	1.640 (1.248–2.156)	*p* < 0.001	1.774 (1.279–2.461)	0.001	1.571 (1.072–2.301)	0.020
High expression group	Reference	Reference	Reference	Reference	Reference	Reference	Reference	Reference
Age								
<60 years			0.814 (0.616–1.077)	0.149	0.734 (0.524–1.029)	0.073	0.846 (0.566–1.266)	0.416
≥60 years			Reference	Reference	Reference	Reference	Reference	Reference
Gender								
Female			1.250 (0.935–1.673)	0.133	1.294 (0.912–1.837)	0.149	1.400 (0.937–2.091)	0.100
Male			Reference	Reference	Reference	Reference	Reference	Reference
Grade								
G1					128.800 (0−1.355E+32)	0.890	48.545 (0−3.450E+35)	0.922
G2					179.261 (0−1.883E+32)	0.883	54.690 (0−3.881E+35)	0.920
G3					163.841 (0−1.722E+32)	0.885	53.358 (0−3.787E+35)	0.920
G4					Reference	Reference	Reference	Reference
Stage								
Stage I					0.649 (0.113–3.736)	0.628	0.402 (0.065–2.498)	0.328
Stage II					0.624 (0.232–1.678)	0.350	0.357 (0.113–1.126)	0.079
Stage III					1.314 (0.653–2.643)	0.444	0.728 (0.318–1.667)	0.453
Stage IV					Reference	Reference	Reference	Reference
T								
T1					0.627 (0.179–2.194)	0.465	0.656 (0.178–2.412)	0.526
T2					0.673 (0.368–1.232)	0.199	0.726 (0.351–1.501)	0.387
T3					0.936 (0.597–1.466)	0.772	0.835 (0.490–1.422)	0.506
T4					Reference	Reference	Reference	Reference
N								
N0					0.274 (0.103–0.731)	0.010	0.347 (0.045–2.694)	0.311
N1					0.229 (0.077–0.68)	0.008	0.329 (0.040–2.707)	0.301
N2					0.518 (0.207–1.299)	0.161	0.735 (0.099–5.452)	0.763
N3					Reference	Reference	Reference	Reference
HPV Status								
No							2.227 (1.003–4.942)	0.049
Yes							Reference	Reference
Radiation therapy								
No							2.958 (1.912–4.577)	*p* < 0.001
Yes							Reference	Reference

*The transcription level of SLC13A4 in HNSCC patients, and those above the median value were defined as the high expression group, while those below the median value were defined as the low expression group.

^a^Unadjusted.

^b^Adjusted for age and gender.

^c^Adjusted for age, gender, grade, stage, T stage and N stage.

^d^Adjusted for age, gender, grade, stage, T stage, N stage, HPV status and radiation therapy.

**TABLE 4:** Relationship between SLC13A4 expression, clinical character and progression free survival in HNSCC patients

Variables	Model 1 (*n* = 499)^a^		Model 2 (*n* = 499)^b^		Model 3 (*n* = 386)^c^		Model 4 (*n* = 331)^d^	
	HR (95% CI)	*p* Value	HR (95% CI)	*p* Value	HR (95% CI)	*p* Value	HR (95% CI)	*p* Value
SLC13A4*								
Low expression group	1.702 (1.275–2.273)	*p* < 0.001	1.702 (1.275–2.273)	*p* < 0.001	1.94 (1.386–2.716)	*p* < 0.001	1.738 (1.195–2.526)	0.004
High expression group	Reference	Reference	Reference	Reference	Reference	Reference	Reference	Reference
Age								
<60 years			0.908 (0.679–1.214)	0.514	0.817 (0.581–1.149)	0.246	0.756 (0.510–1.119)	0.162
≥60 years			Reference	Reference	Reference	Reference	Reference	Reference
Gender								
Female			0.92 (0.662–1.279)	0.621	0.963 (0.658–1.41)	0.848	0.970 (0.638–1.473)	0.886
Male			Reference	Reference	Reference	Reference	Reference	Reference
Grade								
G1					1.755 (0.303–10.176)	0.531	1.361 (0.227–8.149)	0.735
G2					0.686 (0.262–1.797)	0.443	0.573 (0.196–1.672)	0.308
G3					1.352 (0.646–2.828)	0.423	1.030 (0.436–2.431)	0.946
G4					Reference	Reference	Reference	Reference
Stage								
Stage I					267.832 (0−2.816E+47)	0.916	104.121 (0−1.053E+48)	0.932
Stage II					329.130 (0−3.457E+47)	0.913	123.742 (0−1.25E+48)	0.929
Stage III					297.986 (0−3.132E+47)	0.914	107.930 (0−1.091E+48)	0.931
Stage IV					Reference	Reference	Reference	Reference
T								
T1					0.438 (0.098–1.953)	0.279	0.456 (0.101–2.065)	0.308
T2					0.649 (0.356–1.183)	0.158	0.691 (0.344–1.385)	0.297
T3					0.805 (0.498–1.301)	0.376	0.660 (0.380–1.148)	0.142
T4					Reference	Reference	Reference	Reference
N								
N0					0.376 (0.128–1.103)	0.075	0.586 (0.077–4.479)	0.607
N1					0.252 (0.076–0.833)	0.024	0.352 (0.042–2.915)	0.333
N2					0.655 (0.237–1.807)	0.414	1.077 (0.146–7.950)	0.942
N3					Reference	Reference	Reference	Reference
HPV Status								
No							2.155 (0.988–4.701)	0.054
Yes							Reference	Reference
Radiation therapy								
No							1.471 (0.933–2.319)	0.096
Yes							Reference	Reference

*The transcription level of SLC13A4 in HNSCC patients, and those above the median value were defined as the high expression group, while those below the median value were defined as the low expression group.

^a^Unadjusted.

^b^Adjusted for age and gender.

^c^Adjusted for age, gender, grade, stage, T stage and N stage.

^d^Adjusted for age, gender, grade, stage, T stage, N stage, HPV status and radiation therapy.

**TABLE 5:** Relationship between SLC13A4 expression, clinical character and disease specific survival in HNSCC patients.

Variables	Model 1 (*n* = 474)^a^		Model 2 (*n* = 474)^b^		Model 3 (*n* = 371)^c^		Model 4 (*n* = 320)^d^	
	HR (95% CI)	*p* Value	HR (95% CI)	*p* Value	HR (95% CI)	*p* Value	HR (95% CI)	*p* Value
SLC13A4*								
Low expression group	2.171 (1.507–3.127)	*p* < 0.001	2.170 (1.505–3.128)	*p* < 0.001	2.527 (1.635–3.905)	*p* < 0.001	2.361 (1.411–3.951)	0.001
High expression group	Reference	Reference	Reference	Reference	Reference	Reference	Reference	Reference
Age								
<60 years			0.948 (0.665–1.352)	0.769	0.896 (0.587–1.368)	0.611	0.877 (0.521–1.476)	0.622
≥60 years			Reference	Reference	Reference	Reference	Reference	Reference
Gender								
Female			0.983 (0.658–1.468)	0.931	1.009 (0.629–1.619)	0.970	1.049 (0.609–1.806)	0.862
Male			Reference	Reference	Reference	Reference	Reference	Reference
Grade								
G1					0.997 (0.073–13.683)	0.998	0.647 (0.042–9.88)	0.754
G2					0.580 (0.146–2.296)	0.438	0.529 (0.107–2.614)	0.435
G3					1.112 (0.443–2.792)	0.821	0.595 (0.188–1.876)	0.375
G4					Reference	Reference	Reference	Reference
Stage								
Stage I					95.296 (0−3.502E+41)	0.922	32.195 (0−3.166E+44)	0.945
Stage II					125.070 (0−4.585E+41)	0.917	37.021 (0−3.633E+44)	0.943
Stage III					124.483 (0−4.567E+41)	0.917	38.794 (0−3.807E+44)	0.942
Stage IV					Reference	Reference	Reference	Reference
T								
T1					0.461 (0.055–3.863)	0.475	0.522 (0.058–4.724)	0.563
T2					0.525 (0.240–1.150)	0.107	0.466 (0.167–1.300)	0.145
T3					0.991 (0.578–1.697)	0.973	0.878 (0.461–1.67)	0.691
T4					Reference	Reference	Reference	Reference
N								
N0					0.207 (0.066–0.647)	0.007	0.257 (0.031–2.108)	0.206
N1					0.178 (0.049–0.645)	0.009	0.247 (0.028–2.215)	0.212
N2					0.418 (0.149–1.175)	0.098	0.600 (0.079–4.535)	0.620
N3					Reference	Reference	Reference	Reference
HPV status								
No							2.150 (0.759–6.09)	0.150
Yes							Reference	Reference
Radiation therapy								
No							2.324 (1.295–4.17)	0.005
Yes							Reference	Reference

*The transcription level of SLC13A4 in HNSCC patients, and those above the median value were defined as the high expression group, while those below the median value were defined as the low expression group.

^a^Unadjusted.

^b^Adjusted for age and gender.

^c^Adjusted for age, gender, grade, stage, T stage and N stage.

^d^Adjusted for age, gender, grade, stage, T stage, N stage, HPV status and radiation therapy.

### Prognostic Value of SLC13A4 in HNSCC

**FIGURE 3:**
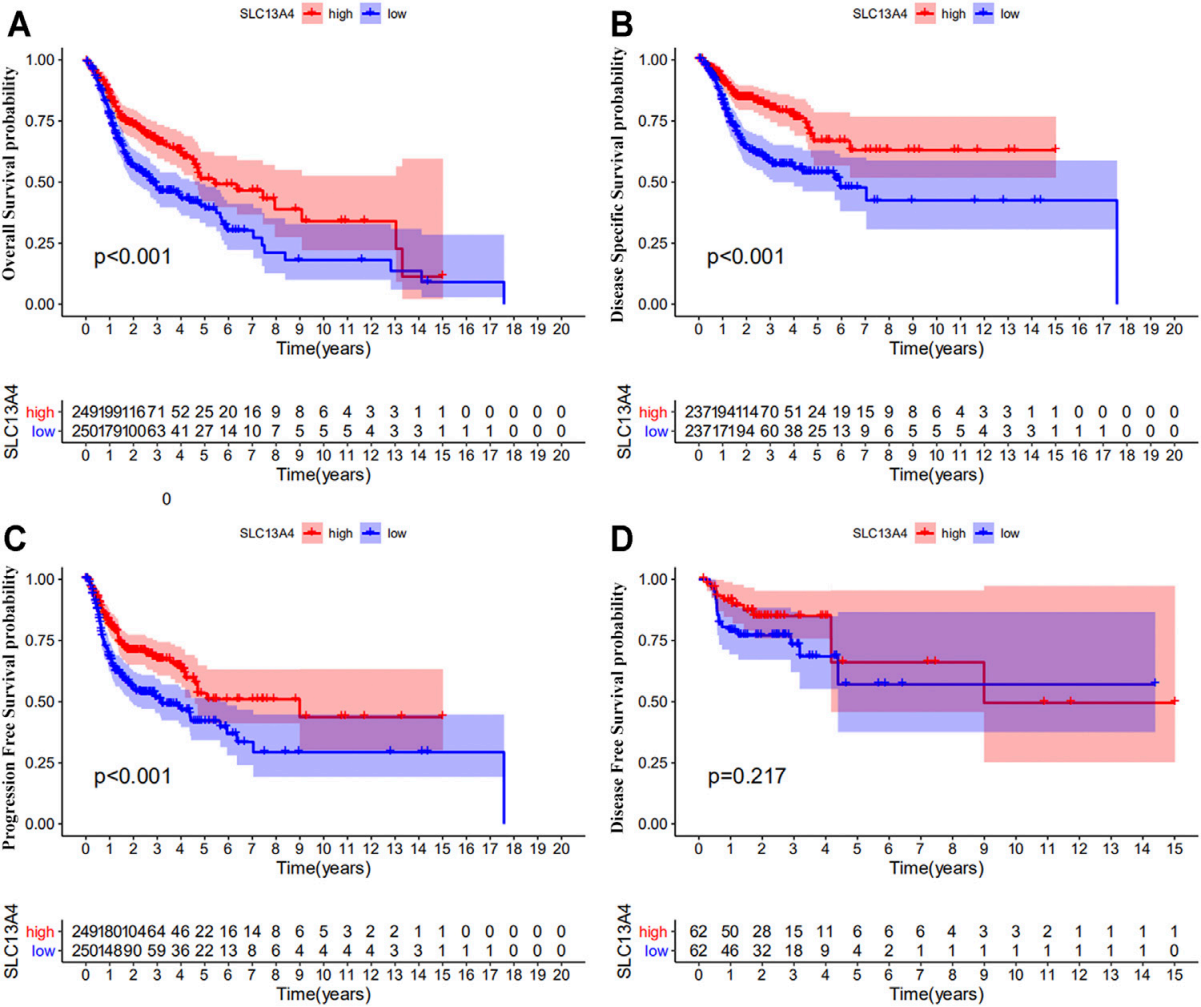
Prognostic significance of SLC13A4 in HNSCC. Kaplan-Meier survival curves of overall survival **(A)**, disease-specific survival **(B)**, progression free survival **(C)** and disease-free survival **(D)** between the high and low expression of SLC13A4 in HNSCC patients from TCGA database.

We theorize that SLC13A4 expression is related to the prognosis of cancer patients. We used TCGA data to evaluate the effects of SLC13A4 expression on the total survival time, disease free survival time, disease specific survival time, and progression-free survival time of HNSCC patients. According to the median mRNA value of SLC13A4 expression, tumor samples were divided into a high expression group and a low expression group. Kaplan-Meier plot analysis was performed and results are shown in Figure 3. Compared with the low SLC13A4 expression group, the patients in the high-expression group had longer overall survival time, progression-free survival time and disease specific survival time (*p* < 0.001), and there was no significant difference in disease free survival time (*p* = 0.217). GEO validation cohort results are shown in Supplementary Figure S1. Compared with the low SLC13A4 expression group, the patients in the high-expression group had longer overall survival time (GSE41613, *p* = 0.010) and recurrence-free survival time (GSE2837, *p* = 0.011). Data from GSE27020 showed that there was no statistical difference in disease free survival time between SLC13A4 low expression group and SLC13A4 high expression group (*p* = 0.092). The findings suggest that SLC13A4 expression level is closely related to clinical characteristics and patients’ survival time, and is potentially of value in the assessment of the prognosis of HNSCC.

### Correlation Between SLC13A4 Expression and Proportion of TIICs and Distribution of TIICs in HNSCC

**FIGURE 4:**
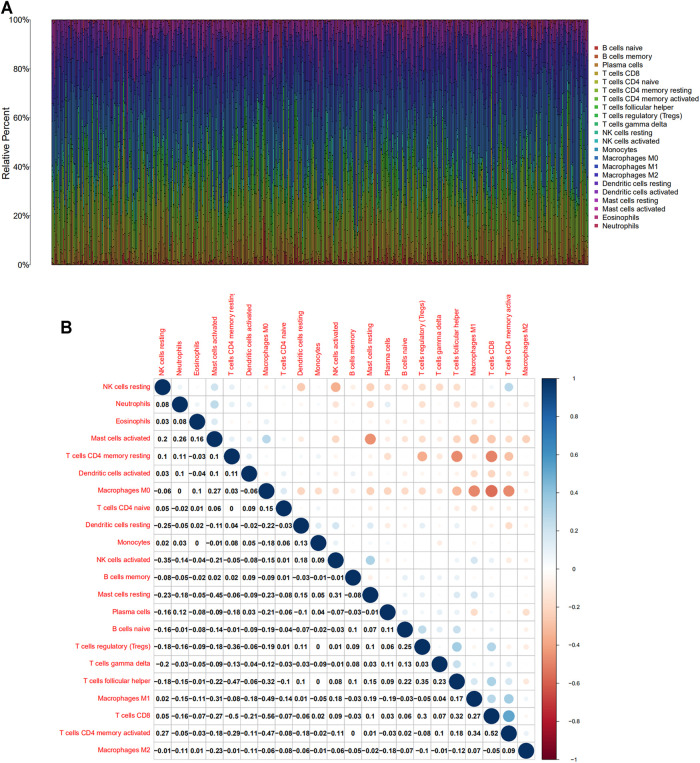
TIC profile and correlation analysis of HNSC samples. **(A)** Barplot showing the proportion of 22 kinds of TICs in HNSC tumor samples. **(B)** Corrplot representing the correlation between 22 kinds of TICs and quantity in each small box, indicating the *p*-value of cells that are correlated with each other. The depth of each tiny color box represents the corresponding correlation value between the two cells, and Pearson coefficient is used for significance test.

**FIGURE 5:**
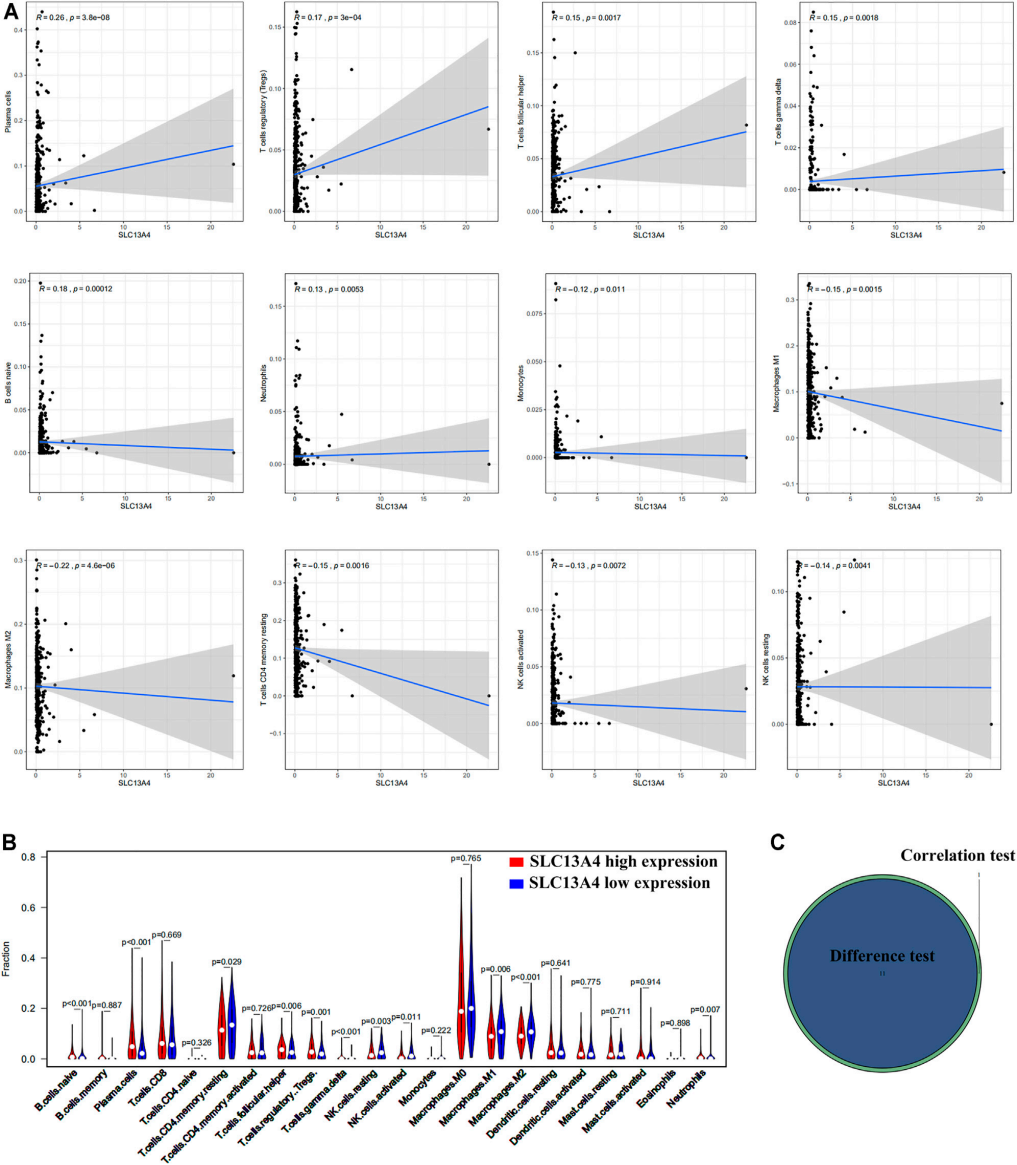
Correlation between TICs proportion and SLC13A4 expression. **(A)** Scatter plot showed the correlation of 12 kinds of TICs proportion with the SLC13A4 expression (*p* < 0.05). The gray line in each plot was fitted linear model indicating the proportion tropism of the immune cell along with SLC13A4 expression, and Pearson coefficient was used for the correlation test. **(B)** Violin plot showed the ratio differentiation of 21 kinds of immune cells in high and low SLC13A4 expression group in HNSC tumor sample, and Wilcoxon rank sum was used for the significance test. **(C)** The Venn diagram shows 11 TICs related to SLC13A4 expression.

Based on Cibersort algorithm, Barplot demonstrated the infiltration of 22 kinds of immune cells in HNSCC tumor samples. The Corrplot showed the correlation among 22 kinds of TIICs in the HNSCC samples. Tumor samples were divided into a high and a low expression group according to the median expression of SLC13A4. The results are shown in Figures 4, 5. The levels of naive B cells (*p <* 0.001), plasma cells (*p <* 0.001), T follicular helper cells ( *p* = 0.007), gamma delta T cells ( *p* = 0.001), regulatory T cells ( *p* < 0.01), neutrophils ( *p* = 0.011) were significantly higher in the high-expression group than in the low-expression group. The levels of resting CD4+ memory T cells ( *p* = 0.023), resting NK cells (*p* = 0.003), activated NK cells ( *p* = 0.007), M1 macrophages (*p* = 0.002), M2 macrophages ( *p <* 0.001)were significantly higher in the low-expression group than in the high-expression group. Correlation analysis showed that the expression of SLC13A4 was negatively correlated with monocytes, M1 macrophages, M2 macrophages, resting CD4+ memory T cells, resting NK cells and activated NK cells, but was positively correlated with neutrophils, plasma cells, T follicular helper cells, gamma delta T cells, regulatory T cells and naive B cells. Furthermore, the expression of SLC13A4 in HNSCC is negatively correlated with a variety of T cell exhaustion markers, including LAG3 ( *p <* 0.001), TIM-3( *p <* 0.001) and CTLA-4 ( *p <* 0.001), indicating its potential mechanism in regulating tumor immunity (Supplementary Table S1). These findings showed that SLC13A4 expression was related to the proportion of TIICs and distribution of TIICs in HNSCC, which further demonstrated that SLC13A4 expression affected the immune activity of HNSCC.

### Correlation Between SLC13A4 Expression and Modulation of Immunity and Tumor Pathways

**FIGURE 6:**
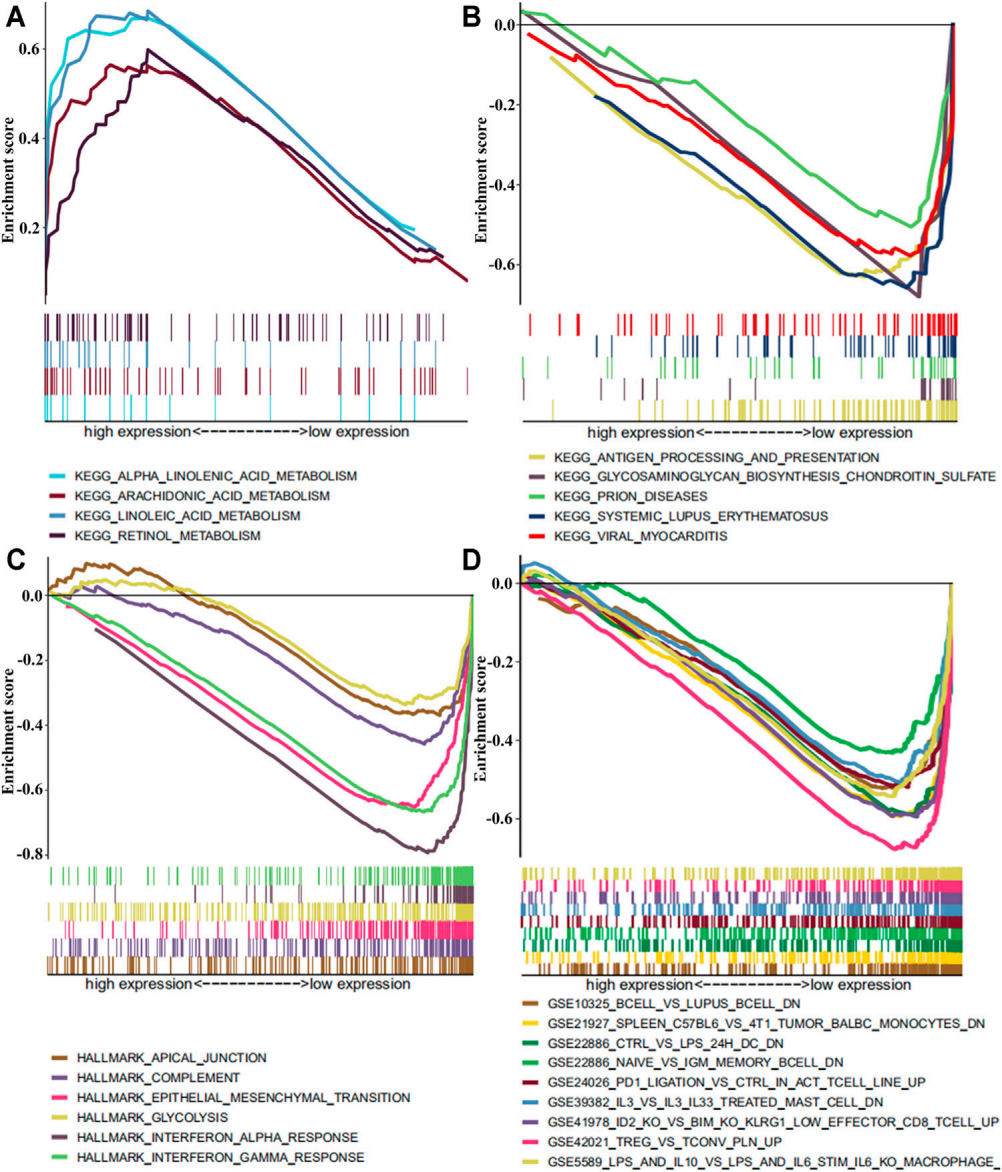
The GSEA enrichment analysis by expression level of SLC13A4 in HNSCC patients. Each row represents a specific set of genes with a unique color. **(A)** Enriched gene sets with high expression of SLC13A4 in KEGG. **(B)** Enriched gene sets with low expression of SLC13A4 in KEGG. **(C)** Enriched gene sets with low expression of SLC13A4 in HALLMARK. **(D)** Enriched gene sets with low expression of SLC13A4 in IMMUNE SIGNITURE and only a few major gene sets were shown (*p* < 0.01 and FDR ≤ 25%).

In order to further understand the potential mechanism of SLC13A4 affecting the prognosis of HNSCC, patients were divided into a high- and a low-expression groups according to the median expression level of SLC13A4 for GSEA enrichment analysis. The results are shown in Figure 6. The genes in low SLC13A4 expression group were principally involved in immunity-related activities, viral diseases, typical tumor pathways and metabolism, including antigen processing and presentation, regulatory T cells, interleukin-family members, viral myocarditis, epithelial mesenchymal transition (EMT), glycosaminoglycan biosynthesis chondroitin sulfate, among others. In the high SLC13A4 expression group, mainly the metabolism-related pathways were enriched, including metabolisms of linoleic acid, retinol, arachidonic acid and alpha linolenic acid. However, there were no enrichment of genome sets involving immune-related activities and typical tumor pathways in the high SLC13A4 expression group, which further suggested that low SLC13A4 expression might be implicated in the process of cancer development and immune activities.

## Discussion

SLC13A4 is a sulfate transporter and is also responsible for the anion transport of selenium and chromium [29]. At present, most studies focused on its roles in severe fetal abnormalities [11], nerve system development [15], and so on. However, sulfate metabolism disorders can cause a variety of diseases and might serve as a predictor for tumor diagnosis [18]. Squamous cell carcinoma is a cancer that arises from particular cells known as squamous cells. Squamous cells are found in the outer layer of skin and in the mucous membranes, which are the moist tissues that form the lining layer of body cavities, such as the airways and intestines. HNSCC is the most common tumor of the head and neck regions, developing in the mucous membrane of the mouth, nose and throat. This study showed that the expression of SLC13A4 was intimately related to the clinical features and prognosis of HNSCC. In HNSCC patients, the expression of SLC13A4 decreased with the increase of tumor pathological grade and clinical stage. The expression level of SLC13A4 in HPV-positive patients was higher than in HPV-negative patients, and the finding was consistent with the phenomenon that the survival rate of HPV-positive patients was higher than that of HPV-negative patients. In this study, only one patient was in M1 stage, so no further analysis was made. Kaplan Meier analysis exhibited that the high expressing SLC13A4 group had longer overall survival time, progression free survival time and disease specific survival time as compared with the low SLC13A4 expression group. On the basis of the aforementioned findings, we are led to believe that that SLC13A4 expression can serve as a predictor of tumor prognosis.

The infiltration of immune cells is one of the characteristic host immune responses to tumor cells and is strongly associated with the development and progression of cancer. Our results also indicated that SLC13A4 expression was significantly correlated with the infiltration of immune cells into HNSCC. SLC13A4 expression was negatively correlated with monocytes, M1 macrophages, M2 macrophages, resting CD4+ memory T cells, resting NK cells and activated NK cells, but was positively correlated with neutrophils, plasma cells, T follicular helper cells, gamma delta T cells, regulatory T cells and naive B cells. These results further confirmed that SLC13A4 level affected the immune activity of TME. SLC13A4 was closely related to the counts of macrophages, M1 and M2, which suggests that the expression of SLC13A4 might be a regulator of macrophage activation and polarization. SLC13A4 was positively correlated with the expression of Tregs, TFH and γδT cells, suggesting that SLC13A4 might regulate the T cell function of HNSCC. Our analysis also showed that SLC13A4 was associated with the degree of immune infiltration and the number of immune cells in HNSCC. It has been reported that the low ratio of M0 macrophages and high expression of Tregs contribute to the favorable prognosis of OS and DFS in HNSCC patients, which is consistent with our conclusion [30]. These findings suggest that SLC13A4 plays an important role in lymphocyte recruitment and control of immune infiltration in HNSCC.

Our GSEA enrichment analysis demonstrated that the genes of in low SLC13A4 expression group were mainly enriched in immunity, viral diseases, typical tumor pathways and metabolism, while genes in high SLC13A4 high expression group were mainly enriched in metabolism-related activities, such as metabolisms of linoleic acid, retinol, arachidonic acid and alpha linolenic acid. However, there was no genomic enrichment involving immunity and typical tumor pathways, suggesting that SLC13A4 might act as an indicator of TME transformation from metabolic dominance to immune dominance. The change of infiltrating immune cell components might be triggered by the growth of tumor tissue, and also affect the development of tumors, such as the tumor immune phenotype could predict their prognosis in patients with squamous cell carcinoma of the oral tongue and with squamous cell carcinoma of the head and neck [31,32]. The change of tumor immune microenvironment provides a new target for studying the development and progression of HNSCC. Immunotherapies aim to enhance the activity of the immune system, thereby eradicating cancer cells. Targeting immune checkpoints is a strategy extensively used in cancer therapy. The expression of SLC13A4 in HNSCC is negatively correlated with T cell exhaustion markers, including LAG3, TIM-3 and CTLA-4. Immune checkpoint inhibitors can block these T cell exhaustion markers signals and have proven their good anti-tumor effects, reverse depletion of T cells, and restore tumor microenvironment and tumor-infiltrating T cell functions [33–35]. These suggest that SLC13A4 might reverse T cell immunosuppression and inhibit tumor immune escape.

In conclusion, this study demonstrated that SLC13A4 might serve as a biomarker of HNSCC. Our study helps clarify the role of SLC13A4 in the prognosis of HNSCC patients and the transformation from metabolic dominance to immune one in TME, and pave the way to future application of the biomarker in the diagnosis and prognostic prediction of HNSCC [28].

## Data Availability

The original contributions presented in the study are included in the article/Supplementary Material, further inquiries can be directed to the corresponding authors.

## Supplementary Material

Supplementary Figure S1 Prognostic significance of SLC13A4 in HNSCC from GEO validation cohorts. Kaplan-Meier survival curves of overall survival in GSE41613 **(A)**, recurrence-free survival in GSE2837 **(B)**, disease specific survival in GSE27020 **(C)**.
