# Supplementary material for: SLC13A4 Might Serve as a Prognostic Biomarker and be Correlated with Immune Infiltration into Head and Neck Squamous Cell Carcinoma
Source: Pathol Oncol Res. 2021 Nov 10;27:1609967. doi: 10.3389/pore.2021.1609967 (PMC8610847; doi:10.3389/pore.2021.1609967)
Supplement: Supplementary file 1 [file Table1.docx]

**Supplementary table 1 :Correlation analysis between SLC13A4 and relate T cell exhaustion markers in TIMER**

|  |  | None | | Purity | |
| --- | --- | --- | --- | --- | --- |
| Description | Gene markers | cor | p Value | cor | p Value |
| T cell exhaustion | PD-1(PDCD1) | -0.08 | 0.0686 | -0.083 | 0.0686 |
|  | CTLA4 | -0.143 | 1.03E-03 | -0.153 | 6.52E-04 |
|  | LAG3 | -0.189 | 1.41E-05 | -0.196 | 1.24E-05 |
|  | TIM-3(HAVCR2) | -0.231 | 1.01E-07 | -0.239 | 8.25E-08 |

None: correlation without adjustment; Purity: correlation adjusted by purity;

Cor：R value of Speraman’s correlation
