# Supplementary figures and images for: SLC13A4 Might Serve as a Prognostic Biomarker and be Correlated with Immune Infiltration into Head and Neck Squamous Cell Carcinoma
Source: Pathol Oncol Res. 2021 Nov 10;27:1609967. doi: 10.3389/pore.2021.1609967 (PMC8610847; doi:10.3389/pore.2021.1609967)

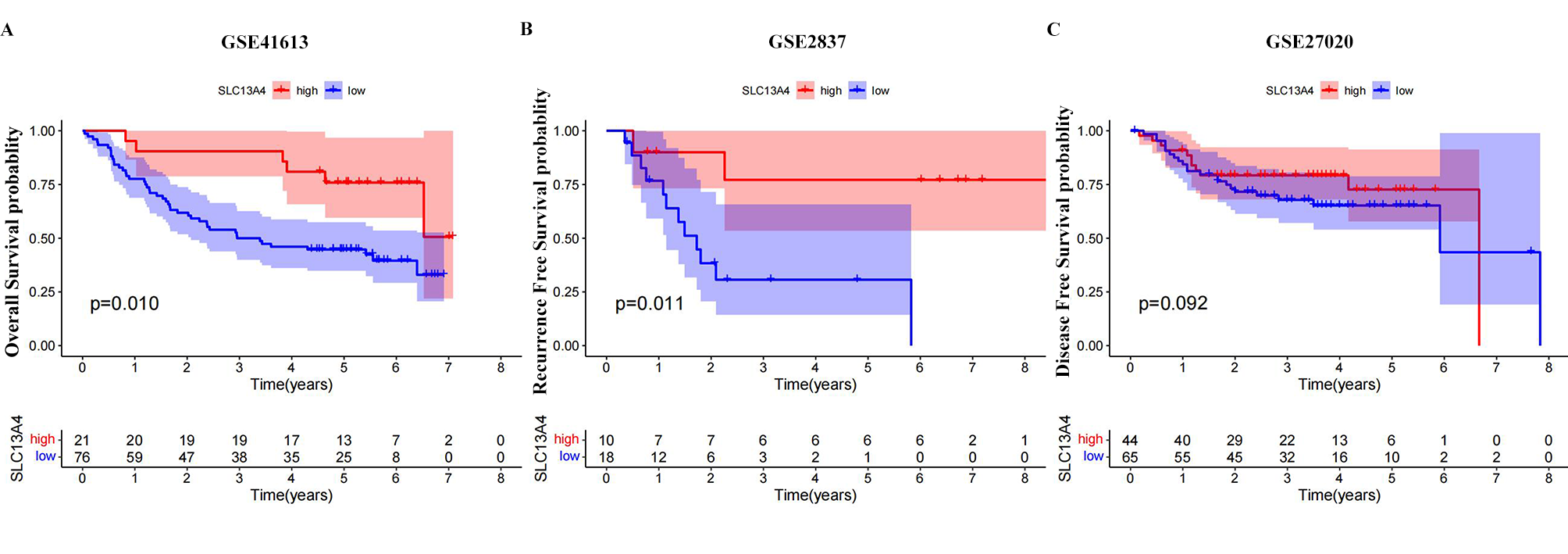

Supplement: Supplementary file 2 [file Image1.TIF]
